# Hesperidin Ameliorates Dexamethasone-Induced Osteoporosis by Inhibiting p53

**DOI:** 10.3389/fcell.2022.820922

**Published:** 2022-04-11

**Authors:** Meng Zhang, Delong Chen, Ning Zeng, Zhendong Liu, Xiao Chen, Hefang Xiao, Likang Xiao, Zeming Liu, Yonghui Dong, Jia Zheng

**Affiliations:** ^1^ Department of Orthopedics, Henan Provincial People’s Hospital, People’s Hospital of Zhengzhou University, Henan University People’s Hospital, Zhengzhou, China; ^2^ Microbiome Laboratory, Henan Provincial People’s Hospital, People’s Hospital of Zhengzhou University, Zhengzhou, China; ^3^ Department of Orthopaedics, Erasmus University Medical Center, Rotterdam, Netherlands; ^4^ Department of Plastic and Cosmetic Surgery, Tongji Hospital, Tongji Medical College, Huazhong University of Science and Technology, Wuhan, China; ^5^ Department of Surgery of Spine and Spinal Cord, Henan Provincial People’s Hospital, People’s Hospital of Zhengzhou University, Henan University People’s Hospital, Zhengzhou, China

**Keywords:** hesperidin, osteoporosis, bioinformatics, KEGG, p53

## Abstract

Osteoporosis is one of the most frequent skeletal disorders and a major cause of morbidity and mortality in the expanding aging population. Evidence suggests that hesperidin may have a therapeutic impact on osteoporosis. Nevertheless, little is known about the role of hesperidin in the development of osteoporosis. Bioinformatics analyses were carried out to explore the functions and possible molecular mechanisms by which hesperidin regulates osteogenic differentiation. In the present study, we screened and harvested 12 KEGG pathways that were shared by hesperidin-targeted genes and osteoporosis. The p53 signaling pathway was considered to be a key mechanism. Our *in vitro* results showed that hesperidin partially reversed dexamethasone-induced inhibition of osteogenic differentiation by suppressing the activation of p53, and suggest that hesperidin may be a promising candidate for the treatment against dexamethasone-induced osteoporosis.

## Introduction

Millions of individuals suffer from skeletal disorders each year, which result in severe morbidity and mortality in the elderly (Kannegaard et al., 2010; Marrinan et al., 2015). Osteoporosis is one of the most frequent skeletal disorders characterized by decreased bone mass and deteriorated bone microarchitecture, leading to skeletal fragility and increased susceptibility to fractures (Golob and Laya, 2015; Qiu et al., 2021). Globally, more than 200 million individuals are affected with osteoporosis, impairing quality of life and imposing a heavy economic burden on individuals and society (Johnell and Kanis, 2006). With the expanding aging population, osteoporotic fractures increase dramatically each year. Currently, the approaches to treat osteoporosis work primarily through inhibiting bone absorption and promoting bone formation (Langdahl, 2021). However, the side effects of anti-osteoporosis drugs pose a huge challenge to the prevention and treatment of osteoporosis in clinical practice (Cuzick, 2001; McClung et al., 2019). As a result, it is extremely important to explore new treatment strategies for osteoporosis.

Hesperidin (hesperetin-7-O-rutinoside), a flavanone glycoside highly abundant in citrus fruits, particularly in oranges, has gained considerable attention in recent years due to its diverse bioactivities (Gattuso et al., 2007). Hesperidin exhibits a variety of potential positive benefits, including antioxidant, anti-inflammatory, anti-atherogenic, and neuroprotective properties, therefore may serve as a promising therapeutic approach for a wide range of disorders (Li et al., 2008; Yamamoto et al., 2013; Semis et al., 2021). Accumulating evidence suggests that hesperidin plays an important role in regulating bone metabolism and bone formation, which may contribute to ameliorate or prevent the onset of osteoporosis. It has been found that hesperidin appears to regulate cell differentiation through Wnt/β-catenin signaling pathway while also influence the mineralization process by increasing the expression of osteogenic gene (ALP, OCN, Osx, and Runx2) in human alveolar osteoblasts (Hong and Zhang, 2020). In a previous study, hesperidin protected bone mass loss by alleviating oxidative stress and inflammation in an ovariectomy rat model (Zhang et al., 2021). Hesperidin could upregulate the expression of osteogenic markers and promote the maturation of bone organic matrix, thus exerting anti-osteoporosis effects *in vitro* and *in vivo* (Miguez et al., 2021). In an experimental rat model, hesperidin intake resulted in bone mass gain in young rats and protected against ovariectomy-induced bone loss in adult rats, as well as reduced oxidative stress and total lipid content (Horcajada et al., 2008). However, the molecular and cellular mechanisms underlying the osteogenic effect of hesperidin are still largely unknown.

**FIGURE 1:**
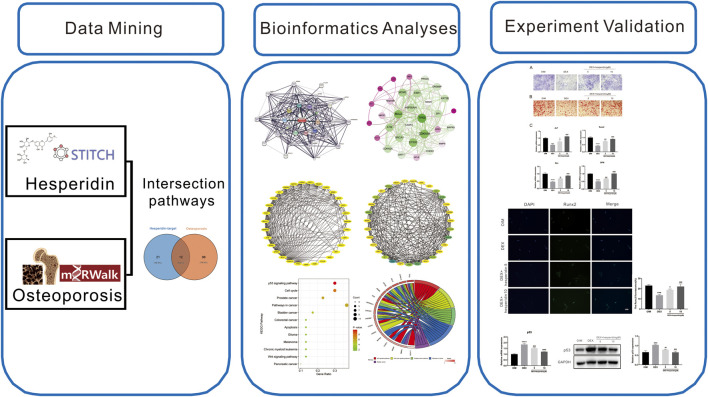
Flowchart of the bioinformatics analyses and experimental validation in this study.

In the present study, we identified the targets genes of hesperidin using the STITCH database, followed by the construction of protein–protein interaction (PPI) network to investigate the protein interactions. In addition, Kyoto Encyclopedia of Genes and Genomes (KEGG) enrichment analysis was carried out to identify pathways that are involved in hesperidin targeted genes and osteoporosis. According to the bioinformatics analyses results, we finally conducted *in vitro* experiments to further investigate the potential molecular mechanisms underlying hesperidin’s anti-osteoporosis effects. The schematic flow chart of this work is shown in Figure 1.

## Materials and Methods

### Regents

Hesperidin was purchased from Herbpurify (Chengdu, China) and dissolved in dimethyl sulphoxide (DMSO). Ascorbic acid phosphate, β-glycerophosphate, and dexamethasone used in this study were purchased from Sigma-Aldrich.

### Cell Culture and Treatment

Bone marrow mesenchymal stem cells (BMSCs) were harvested from C57BL/6 mice and were kept in α-MEM supplemented with 10% fetal bovine serum (FBS) and 1% penicillin/streptomycin according to a previous work (Case et al., 2010). The third to sixth generations of BMSCs were used for our subsequent experiments. To induce osteogenic differentiation, the medium was changed to osteogenic induction medium (OIM) containing 20 mM β-glycerophosphate, 100 nM dexamethasone (DEX), and 50 μM ascorbic acid phosphate once the cells reached subconfluence. BMSCs were co-incubated with DEX (1 μM) and different concentrations of hesperidin (5 and 10 μM) for 7 days or 14 days for further analysis.

### Alkaline Phosphatase and Alizarin Red S Staining

BMSCs were seeded and cultured in OIM on indicated days, then washed with PBS and fixed with 4% paraformaldehyde for 15 min. After 7 days of osteogenic differentiation, alkaline phosphatase (ALP) staining was performed using a BCIP/NBT Kit (C3206, Beyotime) following the manufacturer’s instructions. After 14 days of osteogenic differentiation, alizarin red S (ARS) staining was performed to evaluate the mineralized matrix formation.

### Quantitative Real-Time Polymerase Chain Reaction

The BMSCs were seeded into and cultured in 6-well plates, co-incubated with DEX and different concentrations of hesperidin for 7 days. Total RNA was extracted from the cells using the NucleoZOL Reagent (Machery-Nagel GmbH, Düren, Germany). Next, 1 μg of total RNA from each sample was reverse transcribed into cDNA using the Hifair^®^ II 1st Strand cDNA Synthesis SuperMix for qPCR (YEASON, Shanghai, China). Quantitative real-time polymerase chain reaction (qRT-PCR) was performed on an ABI Stepone plus real-time PCR system (Applied Biosystems, Foster City, CA) with Hieff^®^ qPCR SYBR Green Master Mix (YEASON, Shanghai, China). Relative expression of each gene was analyzed using the 2^−ΔΔCt^ method. The primer sequences were listed in Supplementary Table S1.

### Western Blot

The BMSCs were seeded into and cultured in 6-well plates, co-incubated with DEX and different concentrations of hesperidin for 7 days. Total cell lysates from BMSCs were extracted using RIPA lysis buffer supplemented with protease inhibitors. The protein concentrations were measured with the use of a BCA protein assay kit (Beyotime, Shanghai, China) according to the manufacturer’s protocol. Equal amounts of protein from each sample were then separated by SDS-PAGE gel and subjected to standard western blot procedures. Protein bands were visualized using an ECL kit (BeyoECL Plus, Beyotime Biotechnology) and quantitatively analyzed with Image Lab 3.0 software (Bio-Rad). The antibodies against P53 and GAPDH were purchased from Proteintech (Wuhan, China).

### Immunofluorescence

For immunofluorescence, BMSCs were washed and fixed with 4% paraformaldehyde at room temperature for 30 min, followed by permeabilization with 0.3% Triton X-100 for 10 min. After blocking with 3% BSA for 30 min, the cells were incubated with anti-Runx2 antibody overnight at 4°C. Subsequently, the cells were washed with PBS and incubated with corresponding secondary antibody for 1 h at room temperature, then mounted with DAPI contained fluorescent mounting solution (F6057, Sigma-Aldrich). All images were viewed and photoed with a fluorescence microscope (Olympus).

### Identification of Hesperidin-Targeted Genes and Construction of Protein–Protein Interaction Network

Target genes related to hesperidin were obtained from STITCH (http://stitch.embl.de/) based on the following settings: three shells with a maximum interaction number of 10 for each shell, while the parameter organism was limited to “Homo sapiens” (Szklarczyk et al., 2016). Then, the interactive network map of hesperidin and its targeted genes was constructed, while the degree value in the protein–protein interaction (PPI) network was calculated with the help of the Cytoscape 3.7.2 software. Furthermore, Gephi software was used to establish and visualize a weighted network between hesperidin and targeted genes.

### Identification of Shared KEGG Pathways Involved Both in Osteoporosis and Hesperidin-Targeted Genes

Hesperidin-targeted genes were imported into DAVID database (https://david.ncifcrf.gov/) (Huang et al., 2007), and the KEGG pathway enrichment analysis was carried out to explore the most significantly enriched pathways with a *p*-value of ≤0.05. The miRWalk2.0 database was used to identify KEGG pathways associated with human osteoporosis (Dweep and Gretz, 2015). Subsequently, the overlapping KEGG pathways between osteoporosis and hesperidin-targeted genes were displayed by using Venn diagram webtool (http://bioinformatics.psb.ugent.be/webtools/Venn/).

### Identification of Hub Genes and KEGG Pathways Related to Hesperidin-Targeted Genes

The top five shared KEGG pathways ranked by the smallest *p* values calculated in the KEGG pathway analysis were screened out, followed by graphically visualizing the enrichment information using the bioinformatics platform (http://www.bioinformatics.com.cn/). Hub genes were defined as those genes included in each of the top five shared KEGG pathways.

### Data Analysis

The data was displayed as mean ± standard deviation (SD) and statistically analyzed with GraphPad Prism 8.0 (GraphPad). Statistically differences between two groups were evaluated by Student’s t-test, while comparisons among multiple groups were estimated by one-way analyses of variance (ANOVA). A *p*-value of less than 0.05 indicated statistical significance.

## Results

### Bioinformatics Analyses of Hesperidin-Targeted Genes

**FIGURE 2:**
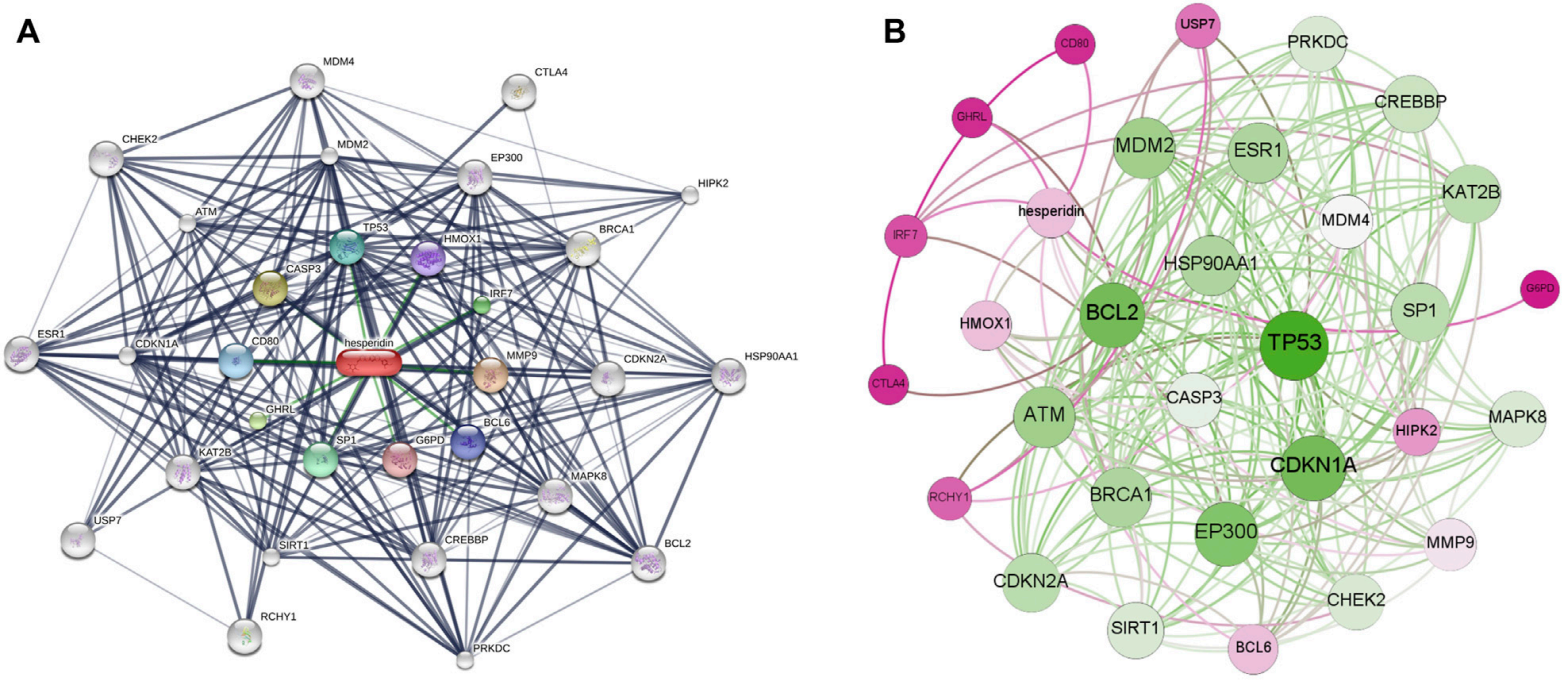
The interaction networks of hesperidin-targeted genes **(A)** Interaction network constructed by Cytoscape **(B)** Weighted interaction network constructed by Gephi.

Based on a three shell limit screening criteria, a total of 30 hesperidin-targeted genes were obtained from online database STITCH. Subsequently, a network map of the interactions between hesperidin and the targeted genes was constructed the STITCH online tool (Figure 2A). Members in the first shell (chemical-protein), including NOS3, PPARG, PTGS2, SIRT1, SIRT3, TP53, AKT1, PTGS1, ESR1 and SIRT5, were considered to be closely related to hesperidin. The second shell (protein-protein) were composed of ATM, BRCA1, EP300, FOXO1, RICTOR, KAT2B, FOXO3, MTOR, CDKN1A, and MDM2, whereas HSP90AA1, CDKN2A, HIPK2, SRC, RCHY1, NCOA3, MAPK8, CREBBP, USP7, and SP1 were found in the third shell (protein-protein and chemical). To facilitate exploration and comprehension of the complicated connections between targeted genes, a visual network based on interaction weights was established (Figure 2B). Further analysis revealed that TP53 was the most highly weighted gene, making it a significant part of the network.

### PPI Network of Hesperidin-Targeted Genes

**FIGURE 3:**
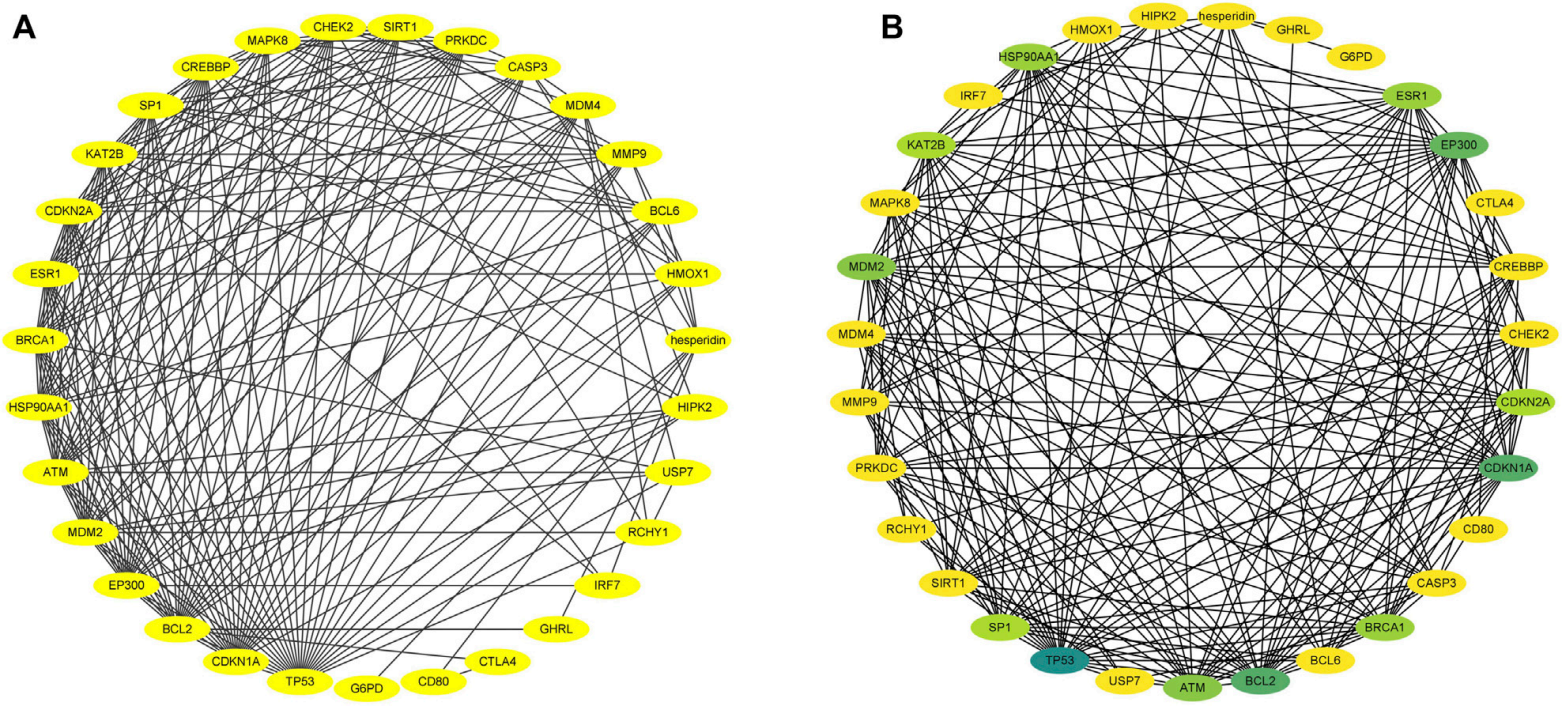
PPI network of hesperidin-targeted genes **(A)** PPI network of hesperidin-targeted genes was constructed by Cytoscape **(B)** PPI network of hesperidin-targeted genes was shown according to degree connectivity. The top ten hesperidin-targeted genes ranked by degree connectivity were displayed as green color.

A visualized PPI network of hesperidin-targeted genes was constructed by using Cytoscape (Figure 3A). Subsequently, the hesperidin-targeted genes were ranked according to degree values (Figure 3B). Due to the fact that CDKN2A, KAT2B and SP1 had the same degree value, the top 10 genes were TP53, CDKN1A, BCL2, EP300, MDM2, ATM, ESR1, HSP90AA1, BRCA1, CDKN2A, KAT2B, and SP1.

### Enrichment Analysis of KEGG Pathways and Identification of Shared KEGG Pathways Between Hesperidin-Targeted Genes and Osteoporosis

**FIGURE 4:**
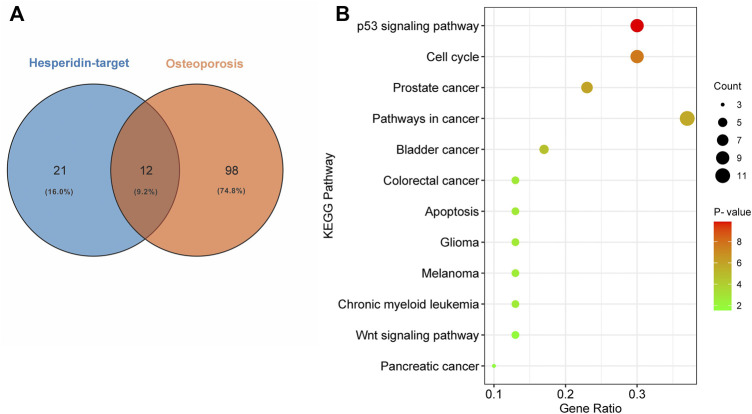
Enrichment analysis of KEGG pathways **(A)** Common shared KEGG pathways between hesperidin-targeted genes and osteoporosis was showed by Venn diagram **(B)** Enrichment information of hesperidin-related KEGG pathways involved in 12 KEGG pathways.

We used DAVID to perform KEGG pathway enrichment analysis. 39 hesperidin-related KEGG pathways were obtained, and 33 KEGG pathways with *p*-value < 0.05 were finally selected. Additionally, 110 KEGG pathways associated with osteoporosis were identified using the miRwalk database. With the help of a Venn Diagram, we were able to identify 12 KEGG pathways that were shared by hesperidin-targeted genes and osteoporosis (Figure 4A). The enrichment information of hesperidin-related KEGG pathways involved in 12 KEGG pathways was shown in Figure 4B. As shown in Table 1, the top five shared KEGG pathways were prostate cancer signaling pathway, pathways in cancer, glioma signaling pathway, p53 signaling pathway, and cell cycle signaling pathway.

**TABLE 1:** Top five KEGG pathways and related genes.

Term	KEGG Pathway	Hesperidin-Targeted Genes	*p*-Value
hsa04115	p53 signaling pathway	CDKN1A, CDKN2A, TP53, MDM2, RCHY1, ATM	1.40E-10
hsa04110	Cell cycle signaling pathway	CDKN1A, CDKN2A, EP300, CREBBP, TP53, MDM2, ATM	2.04E-08
hsa05215	Prostate cancer pathway	AKT1, CDKN1A, HSP90AA1, EP300, CREBBP, TP53, FOXO1, MDM2, MTOR	1.11E-06
hsa05200	Pathways in cancer	FOXO1, AKT1, CDKN1A, EP300, CDKN2A, MDM2, MAPK8, MTOR	1.71E-06
hsa05219	Bladder cancer	CDKN1A, CDKN2A, MDM2, TP53, MMP9	2.01E-05

### Identification of Hesperidin-Targeted Hub Genes

**FIGURE 5:**
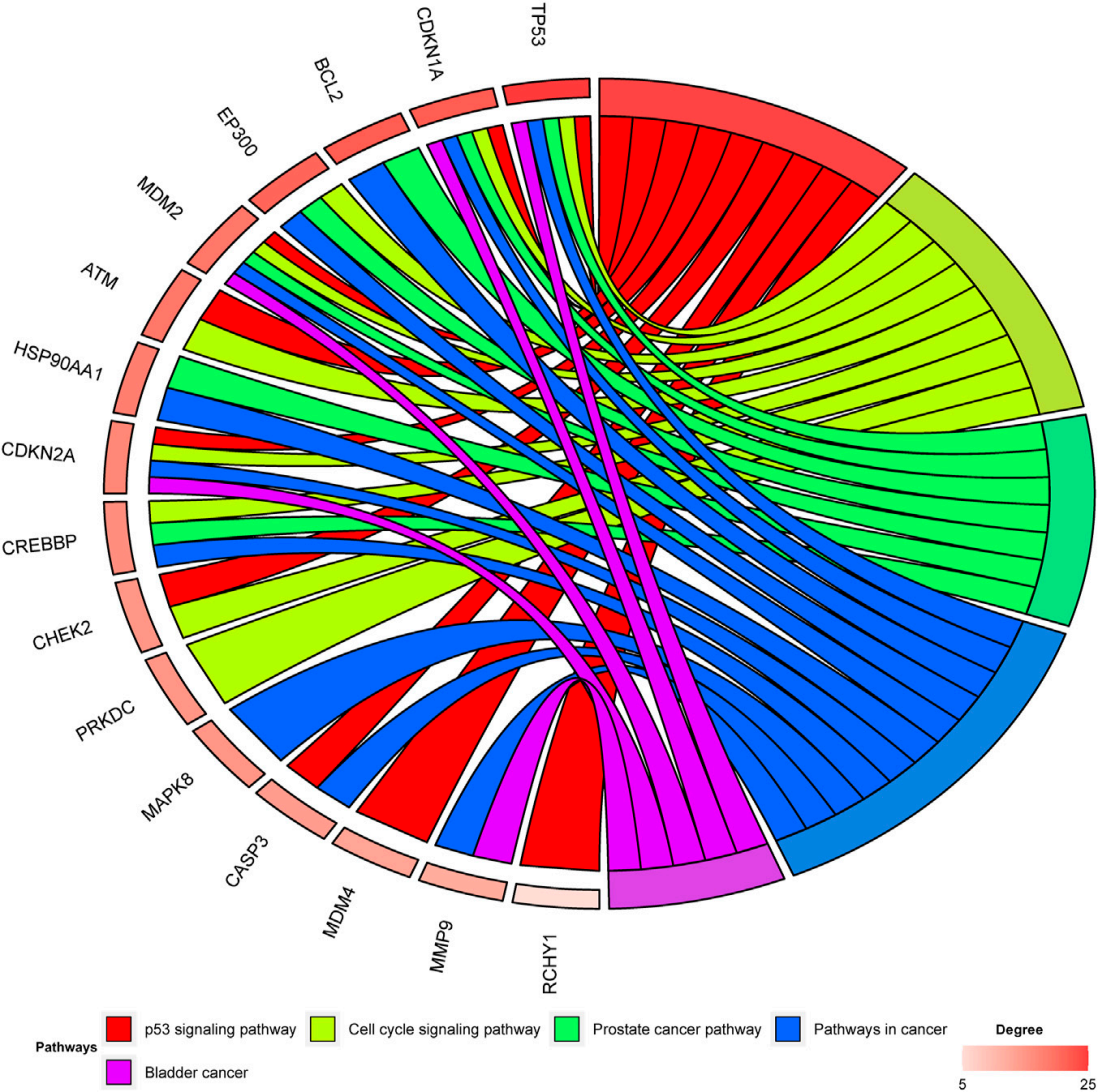
Enrichment analysis of hesperidin-targeted genes. TP53, CDKN1A, and MDM2 were involved in all five shared KEGG pathways. The top three genes by degree were TP53, CDKN1A, and BCL2.

Among the 30 hesperidin-targeted genes, TP53, CDKN1A, BCL2, EP300, MDM2, ATM, HSP90AA1, CDKN2A, CREBBP, CHEK2, PRKDC, MAPK8, CASP3, MDM4, MMP9, and RCHY1 were involved in the top five shared KEGG pathways. The enrichment analysis results of these genes were shown in Figure 5. Importantly, TP53, CDKN1A, and MDM2 were involved in all top five KEGG pathways and were considered as hub genes.

### Hesperidin Partially Reverses Dexamethasone-Induced Inhibition of Osteogenic Differentiation

**FIGURE 6:**
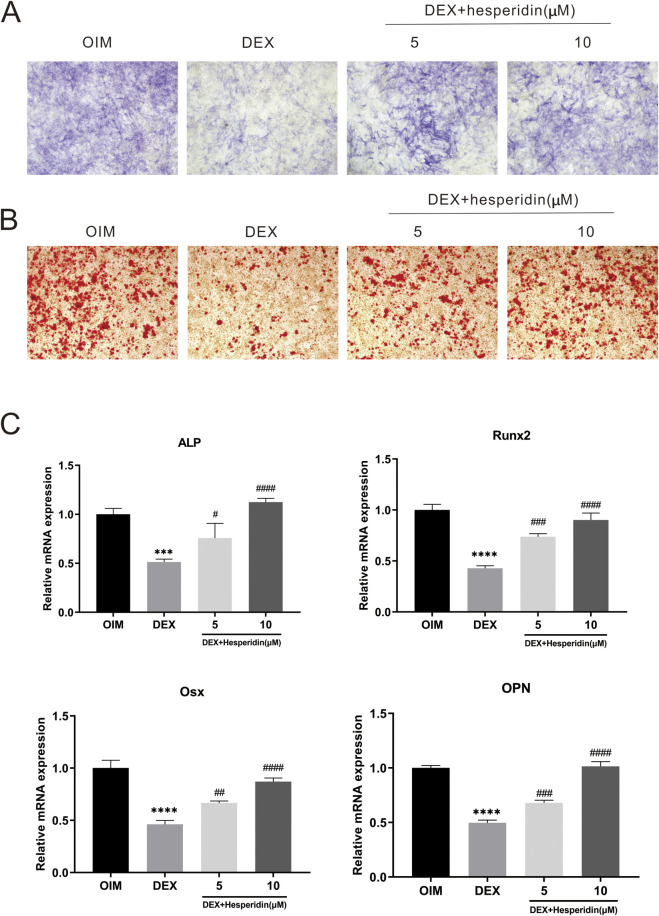
The effect of hesperidin on DEX-induced osteogenic differentiation of BMSCs **(A)** ALP staining was conducted on day 7 **(B)** ARS staining was conducted on day 14 **(C)** The mRNA expression of ALP, Runx2, Osx and OPN were detected by qRT-PCR on day 7. ^***^ *p* < 0.001, ^****^ *p* < 0.0001 vs OIM; ^#^ *p* < 0.05, ^##^ *p* < 0.01, ^###^ *p* < 0.001, ^####^ *p* < 0.0001 vs DEX.

ALP staining and ARS staining results showed that dexamethasone exposure significantly inhibited the osteogenic differentiation of BMSCs, whereas hesperidin partially promoted nodule formation against dexamethasone treatment (Figure 5A and Figure 6B). Furthermore, qRT-PCR analysis results showed that the dexamethasone treatment dramatically downregulated the mRNA levels of osteogenic genes such as ALP, Runx2, Osx, and OPN in BMSCs, but further incubation with hesperidin partially reversed the inhibitory effect of dexamethasone (Figure 6C).

### Hesperidin Partially Reverses Dexamethasone-Induced Osteoporosis by Inhibiting p53 Expression

**FIGURE 7:**
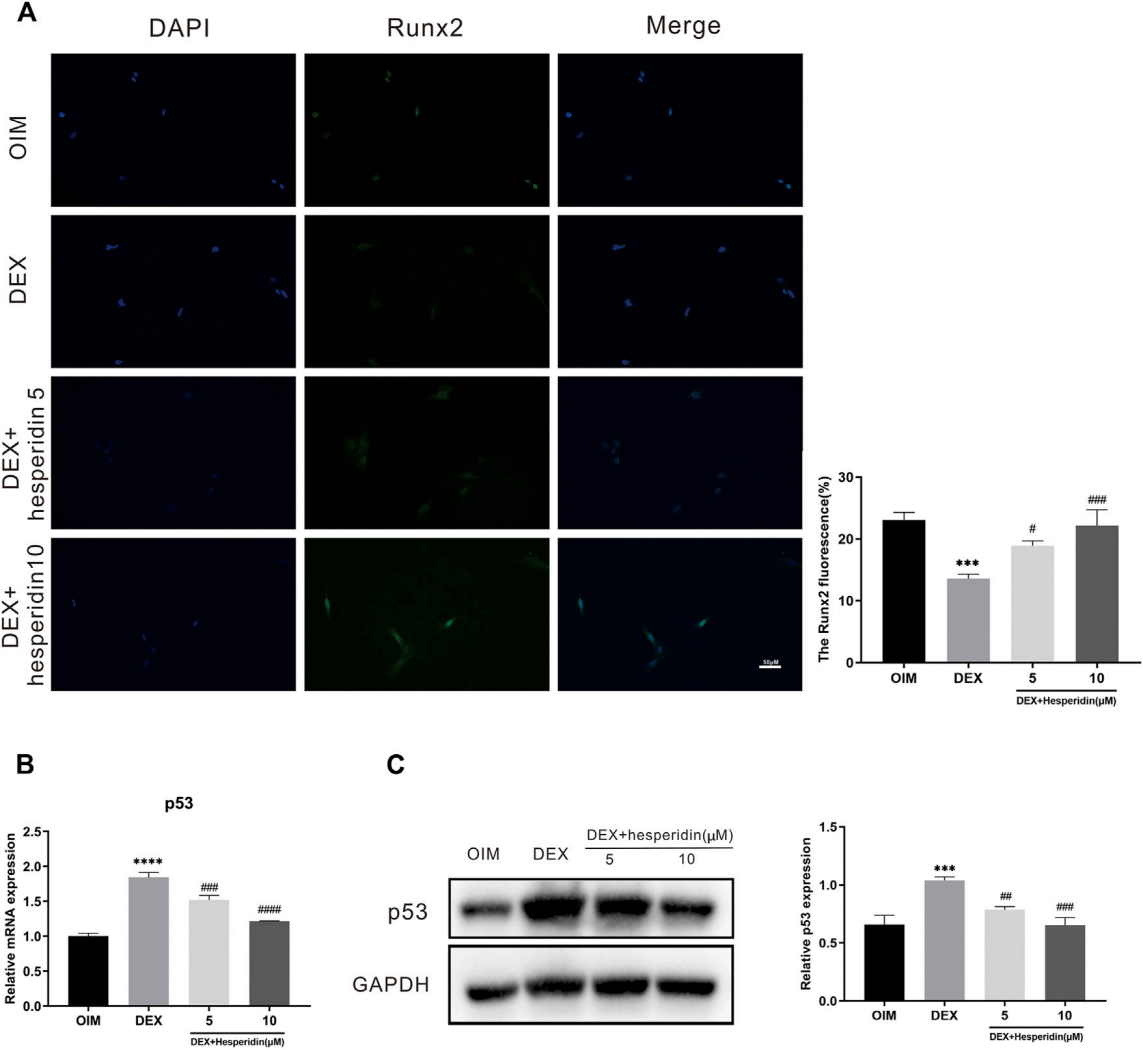
Involvement of the p53 signaling pathway in the regulation of hesperidin **(A)** The typical image of immunofluorescence staining of Runx2. Scale bars = 50 μm **(B)** The relative expression of p53 in the different groups. ^***^ *p* < 0.001, ^****^ *p* < 0.0001 vs OIM; ^#^ *p* < 0.05, ^##^ *p* < 0.01, ^###^ *p* < 0.001, ^####^ *p* < 0.0001 vs DEX **(C)** The level of p53 was detected by western blot.

Immunofluorescence staining results demonstrated that dexamethasone dramatically inhibited the protein levels of Runx2 during the osteogenic differentiation of BMSCs, while higher levels of Runx2 protein expression were observed after hesperidin treatment (Figure 7A). Besides, treated with dexamethasone significantly increased the mRNA level of p53 compared with OIM group, but hesperidin partially inhibited the p53 activation in the dexamethasone group in a dose-dependent manner (Figure 7B). Similar results were also observed regarding the protein expression of p53 detected by Western blot (Figure 7C). Thus, it can be assumed that downregulation of p53 expression alleviated dexamethasone-induced osteogenic reduction.

## Discussion

Osteoporosis is a chronic metabolic bone disorder associated with aging, resulting in functional disability and a decrease in quality of life. As the population ages, a rising number of people currently suffer from osteoporosis substantially. Up to date, there is still no effective treatment for osteoporosis. Furthermore, the mechanisms responsible for osteoporosis remain largely unknown. In the present study, the molecular mechanisms behind hesperidin’s anti-osteoporosis benefits were explored using bioinformatics analyses and *in vitro* studies.

Hesperidin, a flavanone glycoside with a wide range of biological activities, is widely used in the treatment of various diseases. In recent years, emerging studies highlight the importance of hesperidin in the regulation and bone metabolism. Hesperidin has been shown to protect male mice against androgen deficiency-induced bone loss by inhibiting bone resorption and hyperlipidemia (Chiba et al., 2014). It has been reported that hesperidin alleviated diabetic osteoporosis via reducing the expression level of TNF-α and NF-κB in rat bone and increasing the expression of OPN and OCN in serum (Shehata et al., 2017). In addition, calcium supplementation along with hesperidin was effective to improve bone health in postmenopausal women (Martin et al., 2016). However, the exact mechanisms through which hesperidin exerts its anti-osteoporosis effects are still needed to be explored.

In this study, 110 KEGG pathways associated with osteoporosis and 33 KEGG pathways associated with hesperidin-targeted genes were screened out by KEGG pathway enrichment analysis. A total of 13 KEGG pathways were commonly shared by these two groups. Among them, the top five KEGG pathways with the smallest *p*-values were the p53 signaling pathway, Cell cycle signaling pathway, Prostate cancer pathway, Pathways in cancer, and Bladder cancer. The hub genes involved in all five KEGG pathways were TP53, CDKN1A, and MDM2. These findings suggested that hesperidin may exert its biological activity by regulating the p53 signaling pathway.

Glucocorticoids, most commonly dexamethasone, have been widely used to treat a variety of diseases due to their significant anti-inflammatory, immunosuppressive, and metabolic regulator effects (Schacke et al., 2002; Giles et al., 2018). It has been well accepted that there is a strong correlation between long-term glucocorticoids therapy and the development of osteoporosis (Ding et al., 2015; Liu et al., 2018). Growing evidence from *in vitro* and *in vivo* studies shows that glucocorticoids can inhibit osteoblast proliferation and promote its apoptosis, subsequently resulting in the suppression of bone production and growth (Wang Y. et al., 2020; Wang L. et al., 2020).

Consistent with previous findings, the results of the present study suggest that dexamethasone treatment significantly inhibited the osteogenic differentiation of BMSCs following ALP Staining and ARS Staining, as well as downregulated the expression of osteogenic genes such as ALP and Runx2, implying that dexamethasone inhibited osteogenic differentiation of BMSCs *in vitro*. Notably, hesperidin treatment partially alleviated the dexamethasone-induced suppression of osteogenic differentiation, showing the positive effect of hesperidin on dexamethasone-induced bone deterioration.

The p53 tumor suppressor has long been recognized as critical in cancer prevention (Vogelstein et al., 2000; Qin et al., 2018). In recent years, increasing attention has been paid to the role of p53 in skeletal disorders. Several studies conducted *in vitro* have demonstrated that p53 plays a negative role in the differentiation of MSCs(Molchadsky et al., 2010). It was observed that the expression of p53 was increased in patients with osteoporosis, and upregulation of p53 was associated with a decrease bone mass (Yu et al., 2020). Emerging evidence suggests that glucocorticoids can lead to upregulation of p53, causing activation of the p53 signaling pathway (Li et al., 2012; Zhen et al., 2014). Therefore, we investigated whether hesperidin could exert its anti-osteoporosis effects via the p53 signaling pathway. In this study, p53 was identified as the hub gene with the highest degree value in the PPI network. qRT-PCR and western blot analysis confirmed that the expression of p53 was upregulated in BMSCs during osteogenic differentiation after dexamethasone’s treatment. The results indicated that dexamethasone treatment activated the p53 signaling pathway, leading to inhibition of osteogenic differentiation *in vitro*. However, treated with hesperidin partially inhibited both the mRNA and protein level of p53. Thus, when combined with our findings, this evidence suggests that hesperidin may protect against dexamethasone-induced osteoporosis by inhibiting the p53 signaling pathway.

Some concerns and limitations in this study should be acknowledged. Firstly, we did not perform quantitative analysis of the ALP and ARS staining results. Second, the effects of hesperidin on osteogenic differentiation were not investigated while activating or inhibiting the p53 signaling pathway. Furthermore, no *in vivo* evidence was presented regarding the beneficial effects of hesperidin on osteoporosis.

## Conclusion

In summary, our study determined that dexamethasone activated the p53 signaling pathway in BMSCs, causing downregulation of osteogenic markers and suppression of extracellular matrix mineralization during osteogenic differentiation, while hesperidin exerted anti-osteoporosis effects by inhibiting the p53 signaling pathway. Collectively, our study demonstrated that hesperidin could be a potential candidate for the treatment against dexamethasone-induced osteoporosis.

## Data Availability

The original contributions presented in the study are included in the article/Supplementary Material, further inquiries can be directed to the corresponding authors.
