# Correction: Hesperidin ameliorates dexamethasone-induced osteoporosis by inhibiting p53

**DOI:** 10.3389/fcell.2026.1889984

**Published:** 2026-07-23

**Authors:** Meng Zhang, Delong Chen, Ning Zeng, Zhendong Liu, Xiao Chen, Hefang Xiao, Likang Xiao, Zeming Liu, Yonghui Dong, Jia Zheng

**Affiliations:** 1 Department of Orthopedics, Henan Provincial People’s Hospital, People’s Hospital of Zhengzhou University, Henan University People’s Hospital, Zhengzhou, China; 2 Microbiome Laboratory, Henan Provincial People’s Hospital, People’s Hospital of Zhengzhou University, Zhengzhou, China; 3 Department of Orthopaedics, Erasmus University Medical Center, Rotterdam, Netherlands; 4 Department of Plastic and Cosmetic Surgery, Tongji Hospital, Tongji Medical College, Huazhong University of Science and Technology, Wuhan, China; 5 Department of Surgery of Spine and Spinal Cord, Henan Provincial People’s Hospital, People’s Hospital of Zhengzhou University, Henan University People’s Hospital, Zhengzhou, China

**Keywords:** hesperidin, osteoporosis, bioinformatics, KEGG, p53

There was a mistake in Figure 6 as published. Due to authors’ oversight during figure preparation, the wrong ALP staining image of 10 μM hesperidin group was used in Figure 6A. In addition, as Figure 1 presented the overall flowchart of this research, the error in Figure 6 directly affected the accuracy of the information presented in Figure 1. The corrected Figures 1, 6 and their captions appear below.

**FIGURE 1:**
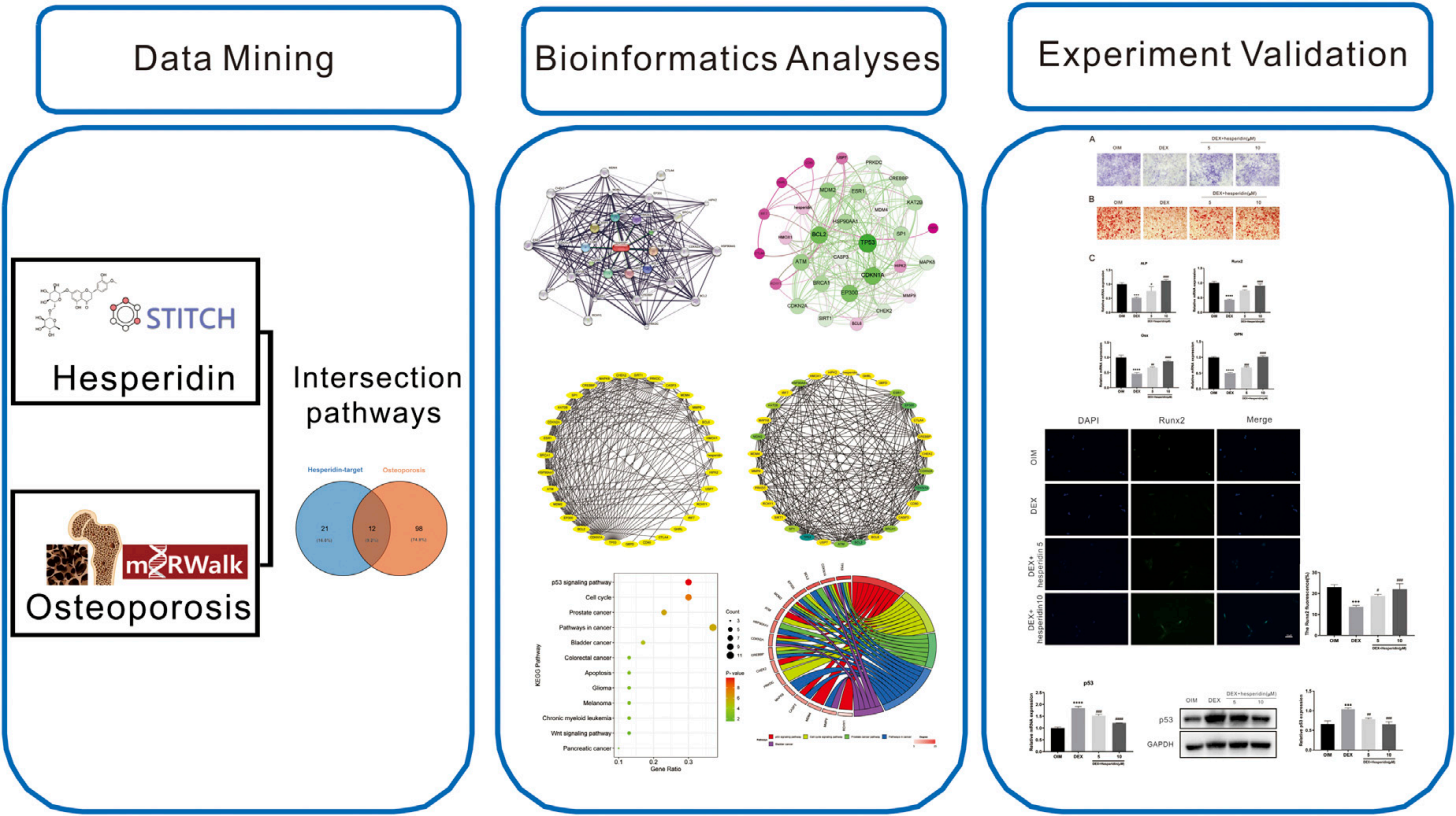
Flowchart of the bioinformatics analyses and experimental validation in this study.

**FIGURE 6:**
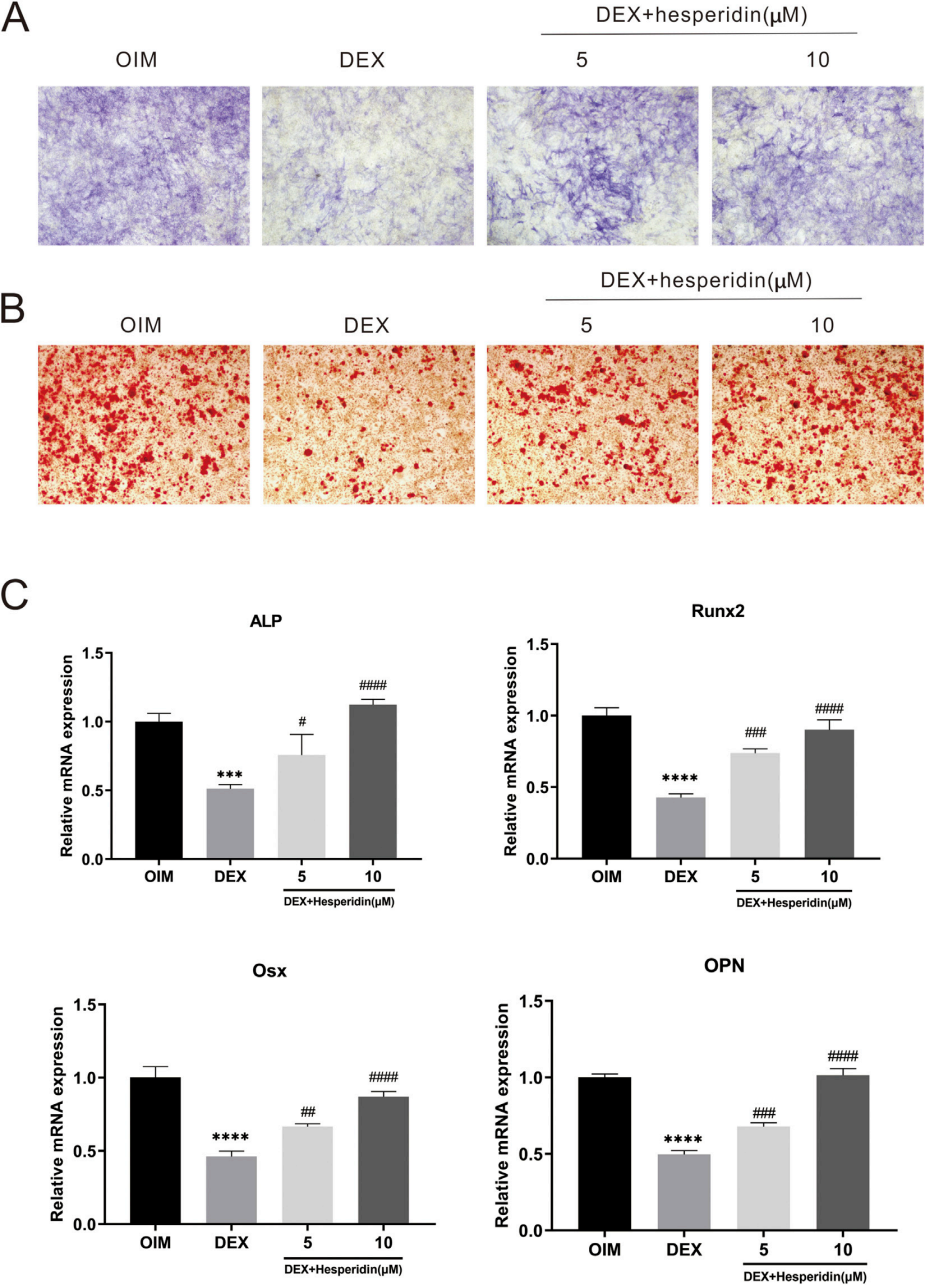
The effect of hesperidin on DEX-induced osteogenic differentiation of BMSCs. **(A)** ALP staining was conducted on day 7. **(B)** ARS staining was conducted on day 14. **(C)** The mRNA expression of ALP, Runx2, Osx and OPN were detected by qRT-PCR on day 7. ^***^ *p* < 0.001, ^****^ *p* < 0.0001 vs. OIM; ^#^ *p* < 0.05, ^##^ *p* < 0.01, ^###^ *p* < 0.001, ^####^ *p* < 0.0001 vs. DEX.

The original version of this article has been updated.

